# Tunnel magnetoresistance angular and bias dependence enabling tuneable wireless communication

**DOI:** 10.1038/s41598-019-45984-5

**Published:** 2019-07-02

**Authors:** Ewa Kowalska, Akio Fukushima, Volker Sluka, Ciarán Fowley, Attila Kákay, Yuriy Aleksandrov, Jürgen Lindner, Jürgen Fassbender, Shinji Yuasa, Alina M. Deac

**Affiliations:** 1 Helmholtz-Zentrum Dresden-Rossendorf, Institute of Ion Beam Physics and Materials Research, Bautzner Landstrasse 400, 01328 Dresden, Germany; 2 Institute of Solid State Physics, TU Dresden, Zellescher Weg 16, 01069 Dresden, Germany; 3 National Institute of Advanced Industrial Science and Technology, Spintronics Research Center, Umezono Tsukuba, 305-8568 Ibaraki, Japan

**Keywords:** Spintronics, Spintronics

## Abstract

Spin-transfer torques (STTs) can be exploited in order to manipulate the magnetic moments of nanomagnets, thus allowing for new consumer-oriented devices to be designed. Of particular interest here are tuneable radio-frequency (RF) oscillators for wireless communication. Currently, the structure that maximizes the output power is an Fe/MgO/Fe-type magnetic tunnel junction (MTJ) with a fixed layer magnetized in the plane of the layers and a free layer magnetized perpendicular to the plane. This structure allows for most of the tunnel magnetoresistance (TMR) to be converted into output power. Here, we experimentally and theoretically demonstrate that the main mechanism sustaining steady-state precession in such structures is the angular dependence of the magnetoresistance. The TMR of such devices is known to exhibit a broken-linear dependence versus the applied bias. Our results show that the TMR bias dependence effectively quenches spin-transfer-driven precession and introduces a non-monotonic frequency dependence at high applied currents. This has an impact on devices seeking to work in the ‘THz gap’ due to their non-trivial TMR bias dependences.

## Introduction

**Figure 1:**
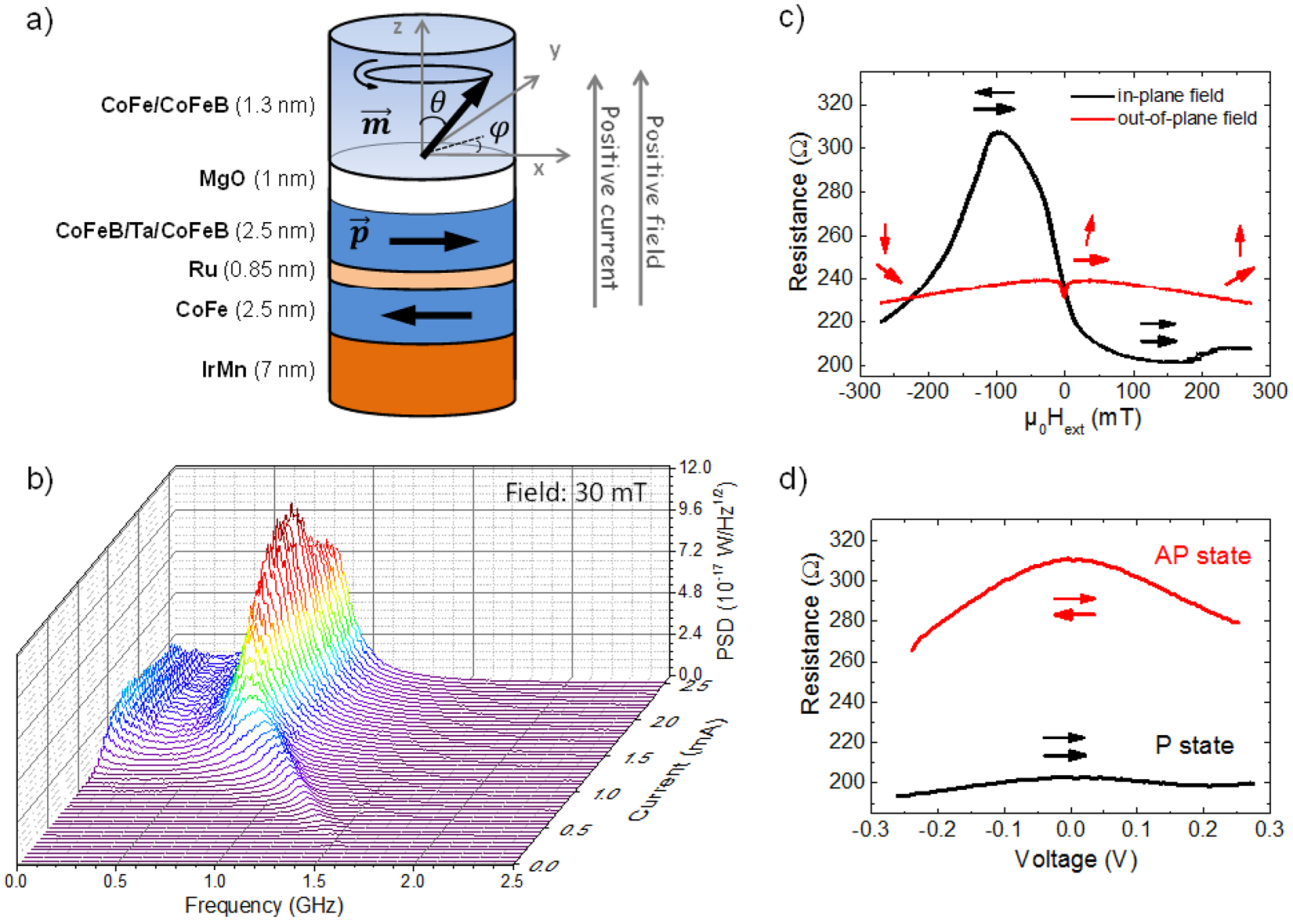
Scheme of the STNO sample, and its static, dynamic and frequency characteristics. (**a**) STNO sample with marked magnetization orientations of the free layer and reference layer (**m** and **p**, respectively), directions of positive applied current and magnetic field, and the coordinate system used in the calculations. The free layer magnetization **m** is depicted precessing at an angle *θ* around the *z*-axis, under the assumption that no other anisotropies are present in the system. In the experiment, an applied DC current, via spin-transfer torque, induces precession of the free layer magnetization, which leads to a time-varying voltage, detected via a spectrum analyzer. (**b**) Frequency spectra versus DC current at a field of 30 mT showing a non-monotonic frequency variation and a decrease of the output power at large currents (above 2.2 mA). (**c**) Magnetoresistance curves measured with in-plane and an out-of-plane magnetic fields (*μ*_0_*H*_*ext*_), exhibit the characteristic behaviour of MTJs with a geometry as shown in (**a**)^7^. The magnetization directions of the free and the reference layers are depicted by the upper and lower arrows, respectively. (**d**) Resistance versus bias voltage, showing a linear decrease in the resistance for the antiparallel (AP) state and an approximately constant resistance for the parallel (P) state.

While initial spin-torque nano-oscillators (STNOs) studies focused on devices with fully in-plane (IP) magnetized magnetic layers^1,2^, hybrid device geometries combining an IP reference layer and an out-of-plane (OOP) magnetized free layer^3,4^ are now the system of choice in view of potential applications^5–9^. Such a system is sketched in Fig. 1(a), with the free layer having an easy axis along the perpendicular to plane (*z*) direction, and the reference layer magnetized along an in-plane direction, defined here as the *x*-axis. This configuration maximizes the output power, reduces the critical current^10^, and can allow for steady-state precession to be excited regardless of applied current or magnetic field history^5,11,12^. State-of-the-art devices, exploiting Fe/MgO/Fe-based MTJs^13,14^, can exhibit output powers orders of magnitude higher (as high as μW)^15,16^ than their fully metallic giant magnetoresistance (GMR) predecessors (limited to a few nW). In comparison to metallic systems, tunnel junction systems have much larger power conversion ratios P_out_/P_in_. For a metallic pillar, P_out_ is of order 1 nW at best, with typical driving currents around 10 mA. Tunnel junctions, on the other hand, have been shown to exhibit much larger P_out_ (up to 2 μW in structures with improved architecture)^16^, with driving currents of about 1 mA, thus yielding a four order of magnitude increase in P_out_/P_in_ compared to their metallic counterparts (see Supplementary Information: Part 1). Simultaneously, these nano-oscillators have a lateral size about 50 times smaller than devices presently used in mobile telecommunication^17^. TMR devices further benefit from low operational current densities of the order of 1 MA/cm^2,6,11,18^, i.e. one order of magnitude lower as compared to the case of metallic spin-valves^4,5^. A device with the same hybrid geometry was recently integrated into phase-locked-loops exhibiting extremely narrow linewidth (less than 1 Hz), which make them suitable for wireless communication applications^19^.

For both GMR and TMR devices, the STT-driven magnetization dynamics can be described by the Landau-Lifshitz-Gilbert-Slonczewski (LLGS) equation^20^:

1 $$\frac{d{\boldsymbol{m}}}{dt}=-\,\gamma {\mu }_{0}({\boldsymbol{m}}\times {{\boldsymbol{H}}}_{{\boldsymbol{eff}}})+\alpha ({\boldsymbol{m}}\times \frac{d{\boldsymbol{m}}}{dt})+\gamma {T}_{\parallel }[{\boldsymbol{m}}\times ({\boldsymbol{m}}\times {\boldsymbol{p}})]+\gamma {T}_{\perp }({\boldsymbol{m}}\times {\boldsymbol{p}}).$$

Here, *γ* is the gyromagnetic ratio, **m** is the unit vector along the magnetization direction, **H**_**eff**_ is the effective magnetic field, *α* is the Gilbert damping constant, **p** is the unit vector defining the direction of the spin-polarization of the current, *T*_||_ is the in-plane (Slonczewski) spin-transfer torque, and *T*_⊥_ is the out-of-plane (field-like) spin-transfer torque.

In GMR devices, ${T}_{\parallel }^{GMR}=\frac{\hslash }{2e}\frac{1}{{M}_{s}{\rm{\Omega }}}g(\theta ,\phi ,\lambda ){I}_{DC}$ (where *M*_*S*_ is the saturation magnetization, Ω is the magnetic volume, *I*_*DC*_ is the applied constant current and *g*(*θ*, *φ*, *λ*) is the asymmetry factor), while ${T}_{\perp }^{GMR}$ is negligible^21^. The underlying mechanism responsible for sustaining dynamics in this hybrid geometry is the spin-transfer torque angular asymmetry, expressed by the asymmetry parameter *λ*^21^. The angular asymmetry of ${T}_{\parallel }^{GMR}$ leads to a *net* Slonczewski torque when integrating over a full precession cycle (as defined by the effective field) for electrons flowing from the free to the reference layer, and therefore allows for compensating the damping torque at sufficiently large currents^5,22^. MTJs have been experimentally shown to exhibit similar dynamics, in spite of the fact that ${T}_{\parallel }^{MTJ}={a}_{\parallel }V$, where *V* is the applied bias voltage and *a*_||_ is a constant known as the in-plane torkance and, therefore, the Slonczewski torque cancels on one precession cycle and cannot counteract the damping. Note that in MTJs the perpendicular STT term, ${T}_{\perp }^{MTJ}={a}_{\perp }{V}^{2}$, is finite (here, *a*_⊥_is the field-like torkance)^23–25^.

In this article, we experimentally and theoretically investigate spin-transfer-driven dynamics in MgO-based MTJs considering the voltage bias dependence of the magnetoresistance and the spin-transfer torques. Experimentally, we observe an unusual, but reproducible curvature of the critical lines in the current-field phase diagram enclosing the region of steady-state dynamics which, to the authors knowledge, has never been reported in similar metallic- or MTJ-based devices. Theoretically, we incorporate the angular dependence^26–28^ and the bias dependence^7,23,29^ of the TMR into spin-transfer torque terms, ${T}_{\parallel }^{MTJ}$ and ${T}_{\perp }^{MTJ}$, and then solve Eq. (1). We find that the angular dependence of the resistance in MTJs, alone, introduces an asymmetry in ${T}_{\parallel }^{MTJ}$ and gives rise to steady-state precession. Moreover, including the bias dependence of TMR correctly reproduces the curvature of the regions of steady-state precession in the experimental phase diagram. Furthermore, we also examine the effect of the bias dependence of TMR on ${T}_{\parallel }^{MTJ}$ and show that it gradually suppresses the induced asymmetry, which ultimately leads to the quenching of dynamics at high bias currents. The analytical formalism presented here allows for the estimation of achievable and realistic device parameter values for driving spin-torque dynamics in MTJ stacks with efficiencies in excess of what can be achieved in GMR devices. Therefore, the TMR ratio, as well as its bias dependence, which are generally not taken into account, are both equally crucial factors governing the performance of MTJ-based STNOs.

## Results and Discussion

**Figure 2:**
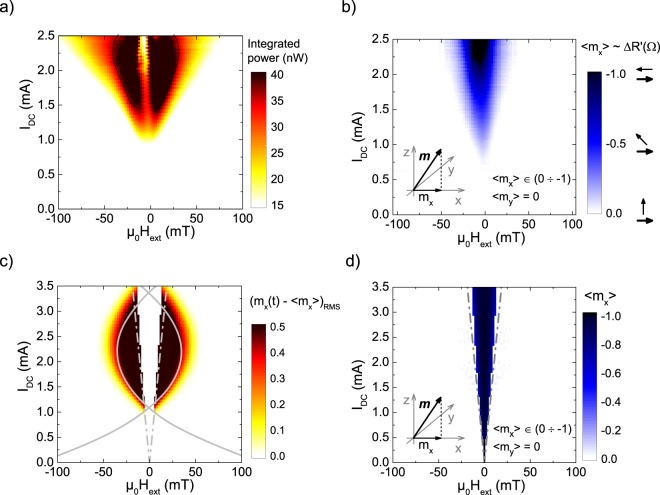
Experimentally (**a**,**b**) and numerically (**c**,**d**) obtained dynamic and static characteristics of a typical nano-oscillator sample. (**a**) Measured output power of the STNO as a function of DC current, *I*_*DC*_, and *μ*_0_*H*_*ext*_. The white dashed line is a guide to the eye marking the strong curvature around the high power region which appears for currents above 2.2 mA. (**b**) Time-averaged projection of the free layer magnetization along the reference layer magnetization (*m*_*x*_), as function of *I*_*DC*_ and *μ*_0_*H*_*ext*_, determined from static resistance measurements. The static in-plane AP state (black region) is stabilized for currents above 2.0 mA. Data in (**a**) is recorded simultaneously with (**b**). (**c**) Computed dynamics intensity of the STNO along *x*-axis as a function of *I*_*DC*_ and *μ*_0_*H*_*ext*_ taking into account a linear dependence of TMR on the applied bias $(\frac{\partial {R}_{AP}}{\partial V}=105\frac{{\rm{\Omega }}}{{\rm{V}}})$. Solid lines show analytically determined critical currents for dynamics and dashed lines show the boundaries marking the stability of the static in-plane AP state. The linear bias dependence of TMR, defined by $\frac{\partial {R}_{AP}}{\partial V}$, is responsible for the curvature of the critical lines. A gradual quenching of dynamics above 2.2 mA is observed with complete suppression of spin-transfer induced dynamics above 3.5 mA. (**d**) Numerically obtained average *m*_*x*_ component as a function of *I*_*DC*_ and *μ*_0_*H*_*ext*_, determining the stability region of the static in-plane AP state. Dashed lines show analytically determined boundaries of the static in-plane AP state.

The experimental geometry and measurement scheme of our STNO devices with marked directions of applied DC current, *I*_*DC*_, and magnetic field, *μ*_0_*H*_*ext*_, is presented in Fig. 1(a). Multilayer films of the following composition: buffer layers/IrMn 7/Co_70_Fe_30_ 2.5/Ru 0.85/Co_20_Fe_60_B_20_ 1.2/Ta 0.2/Co_20_Fe_60_B_20_ 1.2/Co_30_Fe_70_ 0.4/MgO 1/Co_30_Fe_70_ 0.2/Co_20_Fe_60_B_20_ 1.1/capping layers (thicknesses in nm) were grown on Si/SiO_2_ substrates and patterned into nano-pillar devices with the same magnetic volume, but with varying cross sections, by a combination of ultraviolet and electron beam lithography. While all devices show similar trends, here, we present experimental data from a representative device patterned to a (250 × 50) nm ellipse (Figs 1(b,c,d) and 2(a,b)).

Figure 1(b) shows measured STNO frequency spectra versus applied DC current at a 30 mT applied field. We observed a non-monotonic frequency variation and a decrease of the output power occurring for currents above 2.2 mA. The MTJ resistance as a function of *μ*_0_*H*_*ext*_, applied both in the plane of the layers (along the *x*-axis) and along the normal, is shown in Fig. 1(c). The curves confirm that the effective magnetic anisotropy of the free layer, ${\mu }_{0}({H}_{k}-{M}_{s})$, is greater than zero and thus its magnetic easy axis lies out of the film plane^5^. The bias dependence of the resistance, Fig. 1(d), shows a decrease in the resistance for the AP state, with a slope $\frac{\partial {R}_{AP}}{\partial V}=105\frac{{\rm{\Omega }}}{V}$ (for positive voltage driving dynamics), and approximately constant resistance for the P state. For a given range of *I*_*DC*_ and *μ*_0_*H*_*ext*_ applied along the film normal, precession of the free layer magnetization is excited. This yields an oscillatory resistance and, hence, voltage which can be directly detected by means of a spectrum analyzer. The output power of the STNO is then obtained by integration of the fitted peak area (for details, see Supplementary Information: Part 2).

The phase diagram of the observed dynamics as a function of *μ*_0_*H*_*ext*_ (applied along the *z* direction) and *I*_*DC*_ is shown in Fig. 2(a). We find that, similar to metallic devices^3^, spin-torque driven steady-state dynamics can be obtained only for electrons flowing from the free to the reference layer (defined here as positive current), also in agreement with previous reports on MgO-based MTJs^6,18^. The colour code represents the integrated output power, reaching a maximum of 55 nW (obtained for *μ*_0_*H*_*ext*_ = 7 mT and *I*_*DC*_ = 1.9 mA). A clear curvature can be observed at the boundary of the regions of high power steady-state dynamics, denoted with the dashed white line. At low currents, this curvature is quasi-parabolic with increasing external field. Above 2.2 mA the dynamics are gradually quenched, and by extrapolation are assumed to have totally decayed above 2.7 mA. The maximum applied current of 2.5 mA refers to a voltage of 0.775 V (slightly lower than the breakdown voltage of the device, approximately 0.8 V in the AP state).

Magnetoresistance curves are recorded simultaneously with the microwave emission. This allows us to monitor the time-averaged static resistance which is proportional to the projection of the free layer magnetization on the reference layer, as shown in Fig. 2(b). We define the change in resistance Δ*R*′ with respect to the resistance at the same field when using a small probe current which does not stimulate strong dynamics, ${\rm{\Delta }}R^{\prime} {|}_{{\mu }_{0}{H}_{ext}}=R({I}_{DC})-R(0.5\,mA)$. In this way, the value of Δ*R*′ is directly proportional to the average *m*_*x*_ component of the magnetization (for details see Supplementary Information: Part 3). As shown in Fig. 2(b), for field values close to zero, the spin-torque stabilizes the static in-plane AP state^6^. Note also that, according to panels (a) and (b) in Fig. 2, magnetization dynamics are only observable in the presence of finite external fields.

We describe the system analytically using Eq. (1) and introduce the effective field as ${H}_{eff}={H}_{ext}+{H}_{{k}_{\perp }}{m}_{z}$, where *H*_*ext*_ is the applied external field, ${H}_{{k}_{\perp }}$ is the out-of-plane effective anisotropy, and *m*_*z*_ is the projection of the unit vector **m** along *z*.

As experiments are conducted at constant current, the appropriate voltage *V* (which is then introduced into ${T}_{\parallel }^{MTJ}$) is related to *I*_*DC*_ via the following:

2 $$V(\theta ,\phi )={I}_{DC}R(\theta ,\phi )={I}_{DC}\frac{{R}_{P}+\frac{{\rm{\Delta }}{R}_{0}}{2}(1-\,\sin \,\theta \,\cos \,\phi )}{1+\frac{1}{2}|{I}_{DC}|\frac{\partial {R}_{AP}}{\partial V}(1-\,\sin \,\theta \,\cos \,\phi )}.$$

Here, *R*(*θ*, *φ*) is the instantaneous resistance, *R*_*P*_ is the P state resistance, Δ*R*_0_ is the resistance difference between P and AP state close to zero bias, *θ* and *φ* are the angles of spherical coordinate system which define the position of the magnetization vector and, thus, sin*θ* cos*φ* is the projection of the free layer magnetization vector on the direction of the reference layer (i. e., **m** · **p**), and $\frac{\partial {R}_{AP}}{\partial V}$ is the slope of the bias dependence of the AP state resistance.

The following assumptions are made: a linear and quadratic voltage dependence for *T*_||_ and *T*_⊥_, respectively^24,25,29^; a linear bias dependence of the AP state resistance; and a constant P state resistance. We solve Eq. (1) for the instability of static OOP and IP states (see *Methods*). The solutions are plotted as solid lines, dashed lines and by the colour scale in Fig. 2(c), respectively. We also use numerical integration to analyze ensuing dynamical states; the magnitude of the precession motion is defined as the root mean square of the difference of the time-varying component of magnetization along the *x*-axis and its mean value, (*m*_*x*_(*t*) − *m*_*x*_)_*RMS*_. This magnitude is directly related to the experimentally measured output power, as the RF signal from STNOs is to given by the time varying projection of **m** along the magnetization of the reference layer^12,30^, in this case fixed along the *x*-direction (see Fig. 1(a)). The average *m*_*x*_ component of magnetization, obtained numerically, is plotted in Fig. 2(d), along with the analytical critical lines for the static IP states. *m*_*x*_ = −1 corresponds to the AP in-plane state and *m*_*x*_ = 0 corresponds to either alignment of **m** along the *z*-axis or circular precession around the *z*-axis. As in the experiment, stable dynamics occur only when electrons flow from the free to the reference layer. For currents up to around 2.2 mA, the critical current for dynamics scales quasi-parabolically with external field (Fig. 2(c)). However, above this value the region of precession turns back in on itself and dynamics are gradually quenched. Complete suppression of dynamics is achieved for currents above 3.5 mA. To be sure of the underlying cause, we set $\frac{\partial {R}_{AP}}{\partial V}=0\frac{{\rm{\Omega }}}{V}$ and this reproduces what is expected for metallic systems and no curvature is observed^5,12^ (see Supplementary Information: Part 4). While a strict circular cross section was assumed for the analytical calculation, numerical simulations show that the curvature of the critical lines is not affected by varying the shape of the free layer.

The inclusion of the TMR bias dependence $\frac{\partial {R}_{AP}}{\partial V}$ is vital in order to explain the curvature of the critical lines in Fig. 2(a). Comparing the data shown in (a, b) and (c, d) in Fig. 2, the analytical model reproduces qualitatively the main features of the experimental diagram, while numerical data confirms the precession occurs in the areas bound by the critical lines. The results also show that the angular dependence of resistance in MTJs (Eq. (2)) helps to sustain precession in the absence of any intrinsic asymmetry.

**Figure 3:**
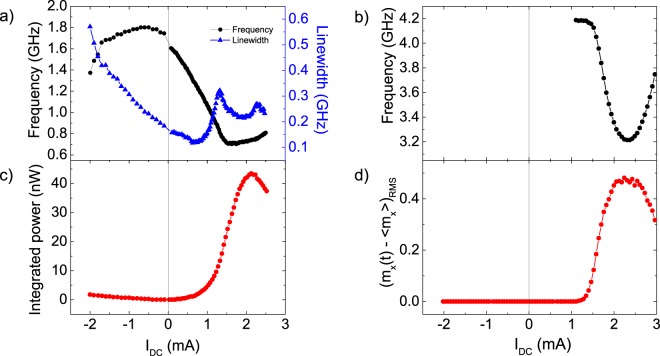
Frequency characteristics of the STNO device. Experimentally and numerically determined frequency and output power of the STNO device at *μ*_0_*H*_*ext*_ = 30 mT. Experimentally measured frequency and linewidth (**a**) and integrated power (**c**) of the STNO as function of *I*_*DC*_. Numerically determined frequency (**b**) and dynamics intensity (**d**) as function of *I*_*DC*_. In both cases, for positive current driving dynamics, we observe an initial rapid decrease in frequency, followed by a region of steady output power and frequency, and, finally a quenching of the output power. There is also an increase of the frequency due to the suppression of the spin-transfer torque asymmetry and ensuing quenching of the precession trajectory.

Further comparison can be made between experiment and numerical results by analyzing the expected precession frequency and output power. The measured frequency and output power as a function of *I*_*DC*_ at *μ*_0_*H*_*ext*_ = 30 mT are shown in Fig. 3(a,c). Numerically obtained frequency and values of (*m*_*x*_(*t*) − *m*_*x*_)_*RMS*_ are shown in Fig. 3(b,d).

According to Fig. 3(a), for positive currents, the first signal is detected at 0.075 mA, at 1.6 GHz, corresponding to an estimated precession angle of 46° (see Supplementary Fig. S4). The frequency decreases with increasing current, reaching a minimum of 0.7 GHz at 1.5 mA, and then starts to increase again with a different slope. For positive current up to around 0.75 mA, the linewidth initially decreases with the current, which indicates that the increasing spin-transfer torque opposes the damping torque. For currents above 1 mA, the increase of the linewidth is a sign of increasing incoherency in the precession^31^, as confirmed by the presence of the 1/f noise (as shown in Supplementary Fig. S5). The maximum linewidth is reached at 1.4 mA, where the estimated precession angle becomes larger than 90° (see Supplementary Fig. S4), indicating that, for currents above 1.4 mA, the macrospin approximation becomes invalid and inhomogeneous dynamics occur. This is further supported by the increase of the linewidth at higher currents, as well as the increasing 1/f tail (see Supplementary Fig. S5). It also has to be pointed out that the minimum frequency and maximum output power are reached at the same current in the simulation (2.25 mA). This is not the case in the experiment, further confirming the breakdown of the macrospin approximation under large applied currents. The frequency tuneability is estimated to be 0.67 GHz/mA between 0.2 and 1.5 mA, while the output power maintains the same order of magnitude as the maximum output power which can be achieved with this specific device. This is similar to values previously reported in the literature for MgO-based magnetic tunnel junctions, which range between 0.1 and 2 GHz/mA^7,17,18,32^.

For negative currents, a measured power is significantly lower (zero in the calculations). We attribute this to thermally-excited ferromagnetic resonance, also known as magnoise^15^. For this current polarity, the in-plane spin-torque acts as damping, yielding a significant increase of the signal linewidth with increasing current is observed. Note that this behaviour, as indeed the linewidth versus current dependence, is similar to the one exhibited by metallic samples^5^.

The analytical model also enables us to determine the dependence of ${T}_{\parallel }^{MTJ}$ on the angle between **m** and **p**, *β*, as well as the previously defined device parameters:

3 $${T}_{\parallel }^{MTJ}(\beta )=\frac{\partial {\tau }_{\parallel }}{\partial V}\frac{{R}_{P}+\frac{{\rm{\Delta }}{R}_{0}}{2}(1-\,\cos \,\beta )}{1+\frac{1}{2}|{I}_{DC}|\frac{\partial {R}_{AP}}{\partial V}(1-\,\cos \,\beta )}\,\sin \,\beta \cdot {I}_{DC}.$$

**Figure 4:**
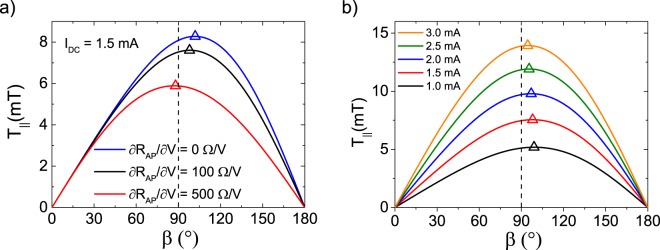
The in-plane component of the spin-transfer torque (*T*_||_) as function of the angle *β* between the free and the reference layers in the STNO (as obtained using Eq. (3)). (**a**) The angular dependence of *T*_||_ for different values of $\frac{\partial {R}_{AP}}{\partial V}$ at *I*_*DC*_ = 1.5 mA. An increase of $\frac{\partial {R}_{AP}}{\partial V}$ results in a reduction of the *T*_||_ angular dependence asymmetry. (**b**) The angular dependence of *T*_||_ for different values of DC current *I*_*DC*_, and $\frac{\partial {R}_{AP}}{\partial V}=100\frac{{\rm{\Omega }}}{{\rm{V}}}$. Increasing the applied current results in an increase of *T*_||_, but simultaneously a reduction of the *T*_||_(*β*) asymmetry. The maximum of each curve is marked with an open triangle and *β* = 90° is marked with a dashed line.

Figure 4 shows the asymmetry of the angular dependence of *T*_||_ (Eq. (3)) for different values of $\frac{\partial {R}_{AP}}{\partial V}$ at an applied DC current of 1.5 mA (Fig. 4(a)), and for different values of *I*_*DC*_ for $\frac{\partial {R}_{AP}}{\partial V}$ of 0, 100 and 500 $\frac{{\rm{\Omega }}}{{\rm{V}}}$ (Fig. 4(b)). The blue line for $\frac{\partial {R}_{AP}}{\partial V}=0\frac{{\rm{\Omega }}}{{\rm{V}}}$ shows the intrinsic spin-transfer torque asymmetry, with the maximum torque at a relative angle of 102°, arising solely from the cosine dependence of the resistance when experiments are conducted at a constant applied current and the in-plane spin-transfer torque scales as the corresponding voltage. Increasing the value of $\frac{\partial {R}_{AP}}{\partial V}$ to 100 $\frac{{\rm{\Omega }}}{{\rm{V}}}$ and 500 $\frac{{\rm{\Omega }}}{{\rm{V}}}$ shifts the maximum of *T*_||_ closer to 90° (98° and 88°, respectively). Indeed, increasing $\frac{\partial {R}_{AP}}{\partial V}$ reduces the TMR amplitude at the considered applied bias and hence counteracts the amplitude of the cosine oscillations of the resistance as function of angle, thereby decreasing the spin-torque asymmetry. For these parameters, we estimate that the asymmetry disappears for $\frac{\partial {R}_{AP}}{\partial V}$ = 330 $\frac{{\rm{\Omega }}}{{\rm{V}}}$, where the resistance of the antiparallel state becomes effectively equal to that of the parallel configuration.

Similar considerations explain the trends observed when analyzing the spin-torque angular dependence for fixed $\frac{\partial {R}_{AP}}{\partial V}$ (100 $\frac{{\rm{\Omega }}}{{\rm{V}}}$ in this case) and different values of the applied current (note a shift of the maximum from 99° to 94° corresponding to the current increase from 1 to 3 mA), see Fig. 4(b). Indeed, one can observe an increase of the magnitude of *T*_||_ with the applied bias, since the magnitude of the torque is proportional to the current. In addition to that, the current is coupled with a reduction of the asymmetry in *T*_||_(*β*), caused by the reduction of the TMR as the bias is increased. Consequently, an increase of the driving current of the STNO brings an enhancement of the output power up to certain current value as the precession angle is increased (reaching a maximum of 0.65 at 2.1 mA, see Fig. 3(d)), above which the loss of asymmetry in *T*_||_(*β*) and TMR become more relevant than the increase of the input power, leading to a decrease in precession angle and finally, the suppression of the dynamics at 3.25 mA.

**Figure 5:**
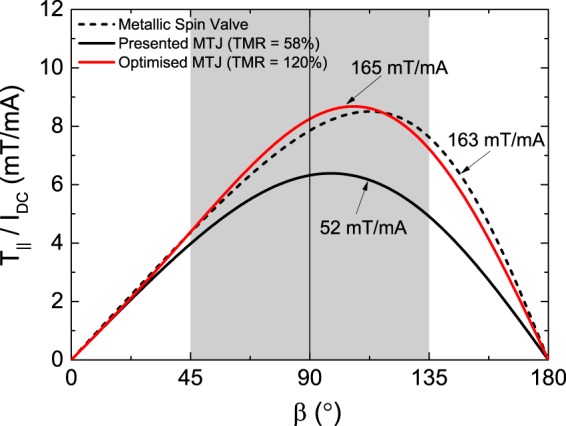
Angular dependence of the spin-transfer torque efficiency $\frac{{T}_{\parallel }}{{I}_{DC}}$. The in-plane spin-transfer torque efficiency $(\frac{{T}_{\parallel }}{{I}_{DC}})$ as a function of *β*, the angle between the free and the reference layer magnetizations. The black solid line is obtained using the parameters of the experimentally investigated MTJ. The red line corresponds to what is expected for a device with an increased TMR ratio of 120%. The dashed line represents the case of a metallic spin valve (using ${T}_{\parallel }^{GMR}$, as described in ref.^5^ with *λ* = 1.5). The corresponding spin-torque efficiencies (expressed in mT/mA) are calculated using the *net* torque over one precession period. A circular trajectory is assumed with an angle of 45° about the *z*-axis. The resulting angle variation over one period is indicated with the shaded region.

Equation (3) is plotted as a function of *β* in Fig. 5 for the experimental device parameters used in the analytical and numerical study (solid black line). The resulting torque displays an asymmetry around 90°, which allows for a *net* torque to be applied to **m** over one precession cycle. The degree of asymmetry is affected strongly by both $\frac{\partial {R}_{AP}}{\partial V}$ and *I*_*DC*_ (see Fig. 4). Higher angular asymmetry of ${T}_{\parallel }^{MTJ}(\beta )$ results in a larger *net* torque, which competes with damping to sustain precession. Larger *net* torque yields a larger magnetization precession angle, which is directly proportional to the output power of the STNO device. With this in mind, we determine the *net* torque over one precession period for an angle of 45°about the *z*-axis (i.e., 45° < *β* < 135°, the shaded region in Fig. 5). This is done by taking the difference of the integrated areas between 45°–90° and 90°–135°, and finally subtracting the damping torque. This then allows for the determination of the efficiency of STT-driven precession, expressed in units of mT/mA. For the experimentally investigated device this efficiency is 52 mT/mA. As a comparison to metallic systems, we also plot the angular dependence of ${T}_{\parallel }^{GMR}$ (using *λ* = 1.5^5^) for the same magnetic free layer, which yields an efficiency of 163 mT/mA (black dashed line in Fig. 5). It can be seen that the efficiency of our MTJ device compares well to that of a metallic system, without the need for high applied currents or large out-of-plane magnetic fields. Equation (3) also allows us to investigate the effect of an increased TMR ratio on the angular dependence of ${T}_{\parallel }^{MTJ}$. The red line in Fig. 5 corresponds to a device with a TMR ratio of 120% (corresponding to Δ*R* = 228 Ω). This value is within the reported range for MgO-based MTJs^33^, and results in an increased efficiency of 165 mT/mA.

## Conclusions

We have shown that the bias and angular dependences of the magnetoresistance ratio in tunnel junctions plays a significant role in understanding spin-transfer driven magnetization dynamics. The angular dependence of the tunnel magnetoresistance introduces an angular asymmetry for the in-plane spin-transfer torque parameter ${T}_{\parallel }^{MTJ}$ which helps to maintain steady-state precession. The bias dependence of the resistance, on the contrary reduces this asymmetry. These two mechanisms, in contrast to fully metallic systems, allow us to tune the asymmetry in ${T}_{\parallel }^{MTJ}$ as a function of current and to control the dynamical response of these devices. The analytical model presented here qualitatively reproduces our experimental findings, including the curvature of the critical lines for dynamics, unique to magnetic tunnel junctions. It also allows us to make predictions for the device parameters which should be optimized in order to fast-track the exploitation of these devices as microwave transmitters and receivers. The bias dependence of the antiparallel state is not readily tuneable, however it has been shown to depend on the barrier properties^34^. A (symmetric or asymmetric) broken-linear TMR bias dependence can be caused by inelastic scattering, asymmetry of elastic tunnelling (reflecting the difference in a quality of the two barrier interfaces), first-order dependence of state density on energy, symmetry of distribution of inelastic tunnelling centers or a combination thereof. However, the assumption of a linear in-plane spin-torque bias dependence is only valid in the first two cases. Therefore, future research should aim at optimizing the barrier of MgO-based tunnel junctions not just for large TMR, but also to control the type of induced defects in order to limit the TMR bias dependence. Last but not least, taking the magnetoresistance bias dependence into account is going to become increasingly important, regardless of the specific materials used in the STNO. Already there is a drive to used STNOs based on Mn-Ga Heusler alloys to enable the realization of technologies beyond 5G. Such materials have been demonstrated to exhibit resonance frequencies of tens^35^ or even hundreds of GHz^36,37^. Their integration into MTJs is a current focus of study for several groups for exactly this reason^38–40^. However, devices based on these compounds display a non-trivial TMR bias dependence, which can be non-monotonic or even change sign as function of the applied voltage^39–41^. The complicated bias dependence of the TMR should be taken into account to enable real world devices based on such materials, and may even explain why efforts to observe magnetization dynamics have been unsuccessful thus far.

## Methods

### Analytical solutions for instability in the OOP and IP static states

Equation (1) is solved for the instability condition of the static OOP state by using the trace and determinant of the Jacobian matrix ***J*** (i.e., when *tr* ***J*** > 0 and *det* ***J*** > 0) at equilibrium positions in the small angle limit *θ* → 0 and *θ* → *π*. The critical lines are given by:

4 $$\begin{array}{lll}{\mu }_{0}{H}_{ext}({I}_{DC}) &  <  & |{a}_{\parallel }{I}_{DC}\frac{{\rm{\Delta }}{R}_{0}-|{I}_{DC}|\frac{\partial {R}_{AP}}{\partial V}{R}_{P}}{\alpha {(2+|{I}_{DC}|\frac{\partial {R}_{AP}}{\partial V})}^{2}}+{a}_{\perp }{I}_{DC}^{2}\\  &  & \times \,(\frac{{\rm{\Delta }}{R}_{0}({\rm{\Delta }}{R}_{0}+2{R}_{P})}{{(2+|{I}_{DC}|\frac{\partial {R}_{AP}}{\partial V})}^{2}}-\frac{|{I}_{DC}|\frac{\partial {R}_{AP}}{\partial V}{({\rm{\Delta }}{R}_{0}+2{R}_{P})}^{2}}{{(2+|{I}_{DC}|\frac{\partial {R}_{AP}}{\partial V})}^{3}})-{\mu }_{0}{H}_{k\perp }|\end{array}.$$

The critical lines for the in-plane AP static state are defined by:

5 $${\mu }_{0}{H}_{ext}({I}_{DC})=\pm \,{a}_{\parallel }{R}_{P}{I}_{DC}.$$

The analytical and numerical solutions shown in Figs 2 and 3 are calculated for the following set of parameters which is realistic in the case of our devices: ${a}_{\parallel }=0.028\,\frac{T}{V}$, ${a}_{\perp }=0.0008\,\frac{T}{{V}^{2}}$, *α* = 0.005, Δ*R*_0_ = 100 Ω, *R*_*p*_ = 190 Ω, *μ*_0_*H*_*k*⊥_ = 120 mT, and $\frac{\partial {R}_{AP}}{\partial V}=105\,\frac{{\rm{\Omega }}}{V}$.

### Numerical simulation

Numerical integration of the LLGS equation (Eq. (1)) was performed using the MAPLE 8 program. We used the same parameters as in the analytical calculations (see above). The simulation enables for the evolution of the position of the magnetization vector under a defined set of parameters to be followed as a function of time. The initial magnetization direction of the free layer was set randomly, in order to take into account the bi-stability regions if they occur. The simulation time was 150 ns, and the final static or dynamic state was defined based on the last 2 ns of the simulation.

## Supplementary information

Supplementary Information

## Data Availability

The datasets generated during and/or analyzed during the current study are available from the corresponding authors on reasonable request.
