# Supplementary material for: Tunnel magnetoresistance angular and bias dependence enabling tuneable wireless communication
Source: Sci Rep. 2019 Jul 2;9:9541. doi: 10.1038/s41598-019-45984-5 (PMC6606570; doi:10.1038/s41598-019-45984-5)
Supplement: Supplementary file 1 — Supplementary Information [file 41598_2019_45984_MOESM1_ESM.pdf]

Tunnel magnetoresistance angular and bias dependence enabling tuneable wireless communication

Ewa Kowalska[1,2]*, Akio Fukushima[3], Volker Sluka[1], Ciarán Fowley[1], Attila Kákay[1], Yuriy Aleksandrov[1,2], Jürgen Lindner[1], Jürgen Fassbender[1,2], Shinji Yuasa[3], Alina M. Deac[1]

[1] Helmholtz-Zentrum Dresden-Rossendorf, Institute of Ion Beam Physics and Materials Research, Bautzner Landstrasse 400, 01328 Dresden, Germany

[2] Institute of Solid State Physics, TU Dresden, Zellescher Weg 16, 01069 Dresden, Germany

[3] National Institute of Advanced Industrial Science and Technology, Spintronics Research Center, Umezono Tsukuba, 305-8568 Ibaraki, Japan

*e.kowalska@hzdr.de

Part 1: STNO efficiency: metallic spin valve versus magnetic tunnel junction

The efficiency of STNOs can be expressed by the power conversion ratio of the output power (emitted by the device) to the input power (required to excite steady-state precession): P_{out}/P_{in}. Typically, for the same lateral size of the nano-pillar, the resistance of a metallic spin-valve is of the order of 10 Ω, and that of an Fe/MgO/Fe magnetic tunnel junction ~100 Ω. The characteristic operation DC currents are experimentally ~10 mA and ~1 mA, respectively. Thus we obtain following input power of these two types of STNOs:

$$P_{in}^{GMR} = RI_{DC}^2 = 10\,\Omega \cdot (0.01\,A)^2 = 10^{-3}\,W \quad (1)$$

$$P_{in}^{TMR} = RI_{DC}^2 = 100\,\Omega \cdot (0.001\,A)^2 = 10^{-4}\,W \quad (2)$$

According to the literature, the output power of STNOs is of order of nW for metallic devices[1], and µW for MgO-based systems[2,3]. Thus, the power conversion ratios are as follows:

$$\frac{P_{out}^{GMR}}{P_{in}^{GMR}} = \frac{10^{-9}\,W}{10^{-3}\,W} = 10^{-6} \quad (3)$$

$$\frac{P_{out}^{TMR}}{P_{in}^{TMR}} = \frac{10^{-6}\,W}{10^{-4}\,W} = 10^{-2} \quad (4)$$

Consequently, the power conversion efficiency of STNOs based on MgO-based magnetic tunnel junctions is four orders of magnitude larger than that of fully metallic devices.

Part 2: STNO output power

The RF signal generated by an STNO device is detected by the spectrum analyzer as a voltage quantity per a defined frequency division. Thus, in order to express the spectral signal in the unit of power, we used the following formula:

$$PSD = \frac{V_{signal}^2 - V_{bg}^2}{Z \cdot RBW}. \quad (5)$$

Here, $PSD\left[\frac{W}{Hz}\right]$ is the Power Spectral Density, V_{signal} is the voltage signal generated by the STNO sample, V_{bg} is the background voltage, Z is the impedance of the circuit (50 Ω), and RBW is the Resolution Band Width (in this case, 3 MHz).

Supplementary Fig. S1 shows an example of data analysis for a single frequency spectrum of the STNO sample measured at +30 mT with a +2.3 mA current. The black line represents the measured spectrum, where the background signal has been previously subtracted. Since in this case a quite significant contribution from the low frequency noise is observed, the overall signal is fitted with Lorentz functions, so that two overlapping peaks can be distinguished: the main mode peak (red curve) and the low frequency noise (green curve). The total output power of the spin-torque oscillator is defined as the area under the red curve over the whole frequency. The power integration was conducted using a Matlab script based on the formula displayed in the inset of Supplementary Fig. S1.

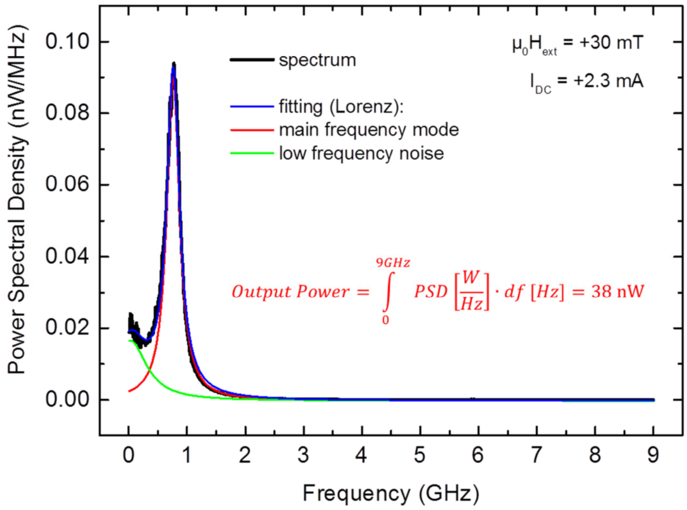

Supplementary Figure S1. An example of data analysis. The output power of spin-torque nano-oscillators is defined as an integral of the Power Spectral Density of the main frequency mode (red curve) over the frequency. Inset formula: PSD - Power Spectral Density, df - frequency step (in our case df = 9 MHz).

Part 3: Static resistance at dynamical states

Simultaneously with the frequency spectra, we measured the static resistance versus field for every current value, shown in Supplementary Fig. S2. The magnitude of the TMR is directly proportional to the relative orientation of the magnetizations of the free and the reference layers, reaching its maximum for the AP state and minimum for the P state. Since the orientation of the reference layer magnetization is fixed, when dynamics are excited, the magnitude of the static resistance gives information about the x-component of the average position of the magnetization in the free layer (i.e., the position of the precession axis).

Supplementary Fig. S2 shows the evolution of magnetoresistance curves with the applied current. We observe here a reduction of the base resistance with increasing current, which results from the TMR bias dependence (see Fig. 1 (d) in the Main Text). For currents up to 0.8 mA, the TMR curve is similar to the curve measured at out-of-plane applied fields for a small probe current of 0.01 mA (see the red curve in Fig. 1 (c) in the Main Text). While approaching zero-field, we observe a dip in the resistance, which occurs due to the canting of the free layer magnetization, induced by an in-plane shape anisotropy of the nano-pillar (note that cross section of the nano-pillar is elliptical), combined with a slight parallel interlayer coupling with the reference layer. For currents above 0.8 mA, where the current density is high enough to drive magnetization dynamics, we observe an increase of the resistance close to zero-field, indicating a gradual tilting of the precession cone toward the in-plane AP orientation (i.e., $-x$ direction in Fig. 1 (a) in the Main Text).

Since the static resistance is proportional to the projection of the free layer magnetization on the magnetization vector in the reference layer, for every applied current, we calculate the change in resistance $\Delta R'|_{\mu_0 H_{ext}} = R(I_{DC}) - R(0.5\ mA)$ with respect to the resistance at the same field when using a small current I_{DC} = 0.5 mA, which does not stimulate strong dynamics (see Fig. 3 (c) in the Main Text). The value of ΔR_0 is directly proportional to m_x. The resistance change ΔR_0 and its equivalent magnetic static state (expressed with averaged m_x component of the magnetization in the free layer) are presented with the colour plot in Fig. 2 (b) in the Main Text. At small fields (0-30 mT), the average position of the magnetization tilts towards the antiparallel configuration (blue region); it finally reaches the AP state for currents above 2 mA (black region). This leads to the preliminary conclusion that the gap in the dynamics at small field, observed in diagrams in Fig. 2 (a), is actually an effect of the stabilization of the static in-plane AP state, under the influence of the spin-transfer torque, which favours the AP state for this current configuration.

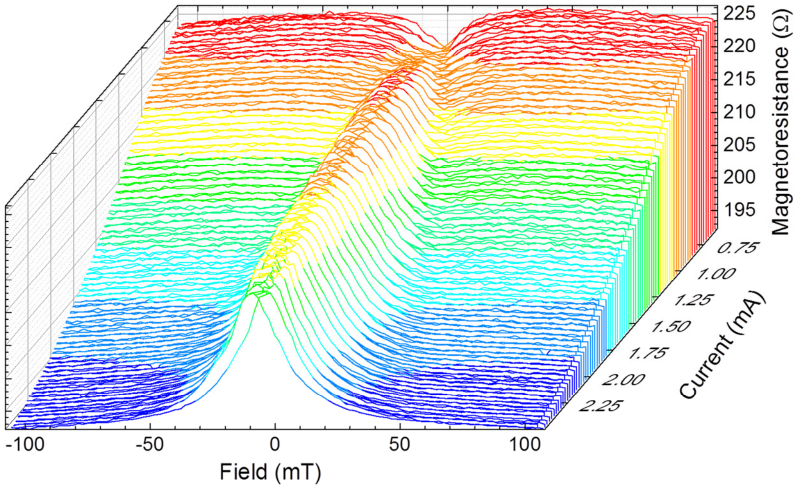

Supplementary Figure S2. Static magnetoresistance versus field for different current values. We observed a general decrease of the overall resistance with the current (due to the TMR bias dependence) and an increased resistance in the small field range (directly proportional to the magnitude of the applied current) indicating a gradual tilting of the precession cone toward the in-plane antiparallel direction.

Part 4: Steady-state dynamics for $\frac{\partial R_{AP}}{\partial V} = 0\ \frac{\Omega}{\mathrm{V}}$

In order to clarify the influence of the TMR bias dependence on the dynamical phase diagram of our STNOs, we initially performed analytical and numerical integrations of the Landau-Lifshitz-Gilbert-Slonczewski (LLGS) equation for the case of $\frac{\partial R_{AP}}{\partial V} = 0\ \frac{\Omega}{\mathrm{V}}$ (i.e., no TMR bias dependence), for currents ranging from -3.5 to 3.5 mA (in 0.05 mA steps) and fields between -100 and 100 mT (with 2 mT increments). Supplementary Fig. S3 (a) displays the critical lines corresponding to the onset currents for precession (solid lines), as well as the quenching currents where the magnetization switches from a precessional state to an in-plane static state (dashed lines), as determined from analytical calculations (see *Methods* in the Main Text). The colour contrast marks the amplitude of the oscillations of the free layer magnetization along the direction defined by the magnetic moment of the reference layer, as obtained from numerical integrations of the LLGS equation. Consequently, the colour contrast should correlate directly with amplitude of the output signal that would be measured in an experiment conducted on such a device. The results are consistent with previous studies on metallic systems, where the intrinsic spin-torque angular dependence asymmetry was included[4]. Turning on the bias dependence of TMR, $\frac{\partial R_{AP}}{\partial V} > 0\ \frac{\Omega}{\mathrm{V}}$, the onset currents for precession exhibit a distinct curvature versus the applied field (see Supplementary Fig. S3 (b)), which has not been previously observed in metallic structures and thus constitutes a distinct signature for MTJs. Meanwhile, the quenching currents where the magnetization transits from steady-state precession to static in-plane states still depend linearly on the applied field (see dash-dot lines in Supplementary Fig. S3 (b)).

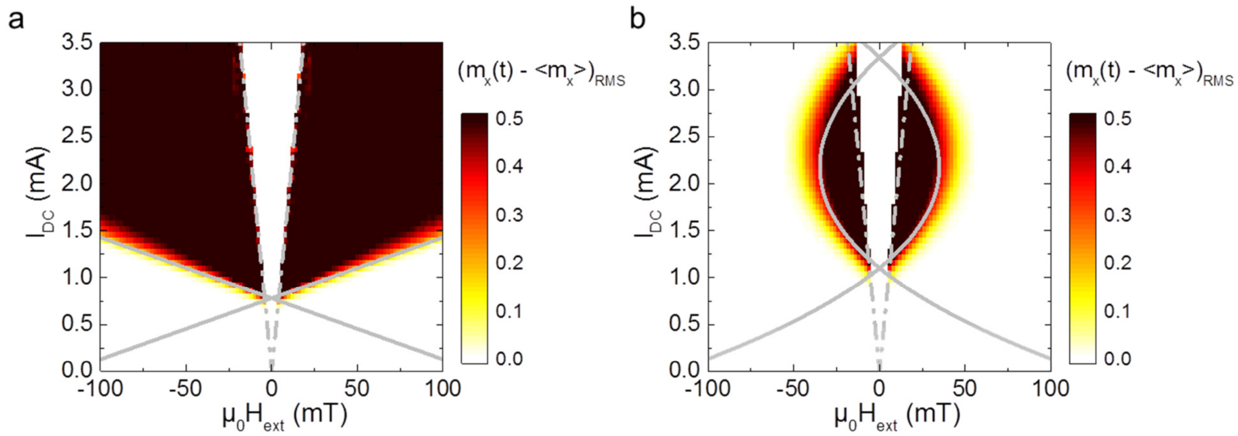

Supplementary Figure S3. Root mean square of the difference between the instantaneous unit magnetic moment along the x-axis (i.e., along the magnetic moment of the reference layer) and its mean value as a function of I_{DC} and $\mu_0 H_{ext}$. (**a**) $\frac{\partial R_{AP}}{\partial V}$ = 0 $\frac{\Omega}{V}$, and (**b**) $\frac{\partial R_{AP}}{\partial V}$ = 105 $\frac{\Omega}{V}$. The plots are limited to positive currents, as no dynamics was observed for negative bias, although the full current range was considered for the calculations.

Part 5: Linewidth and details of spectral features

We estimate the magnetization precession angle θ from the experimentally obtained frequency as a function of I_{DC}, shown in Fig. 3 (a) in the Main Text, using the following formula:

$$\theta = arccos\left(\frac{\frac{2\pi f}{\gamma} - B_{ext}}{B_{k\perp}}\right). \qquad (6)$$

Here, f is the precession frequency, $\gamma = 1.76 \cdot 10^{11} \frac{rad}{s \cdot T}$ is the gyromagnetic ratio, B_{ext} = 30 mT is the external field, and $B_{k\perp}$ = 120 mT is the effective out-of-plane anisotropy.

The derived angle is plotted in Supplementary Fig. S4. The increase of the angle above 90° indicates that the macrospin approximation breaks down above ~ 1 mA. A similar behavior was obtained in metallic systems[1]. This interpretation is further supported by the increase in 1/f noise (see Supplementary Fig. S5), occurring above this bias. 1/f noise is indicative that the dynamics are becoming increasingly less coherent[5].

It is also worth to note that equation (6) is only valid when assuming a constant precession angle θ for a given frequency value (the same approximation we also used in our analytical calculations, see *Methods* section in the Main Text). In the real system, as well as in the macrospin simulation, the precession angle can be assumed as constant only for low currents. For higher applied currents, the magnetization precession trajectory deviates from circular shape and becomes more quasi-elliptical.

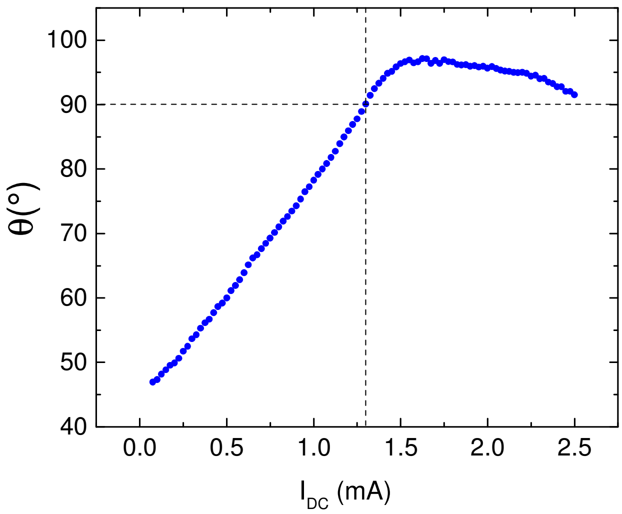

Supplementary Figure S4. Precession angle versus applied current for an applied field of 30 mT estimated using equation (6) from experimentally obtained frequency shown in Fig. 3 (a) in the Main Text.

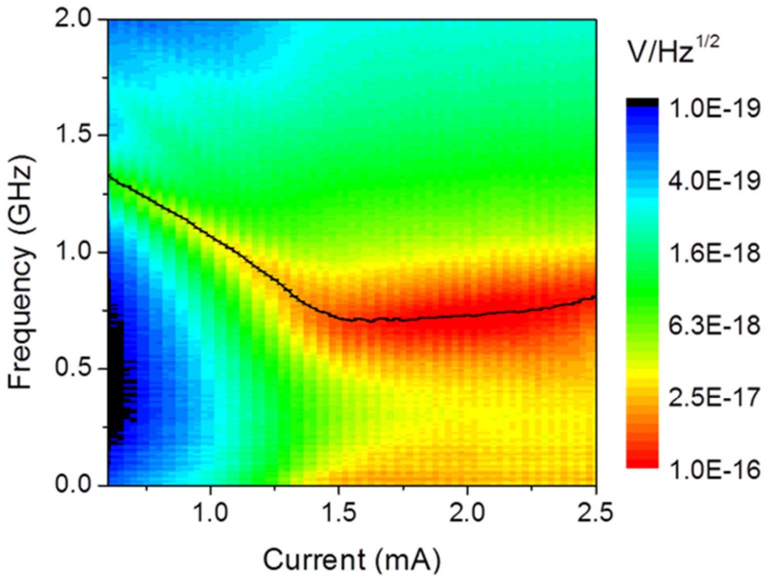

Supplementary Figure S5. Full spectra showing the increase in 1/f noise at the same applied current as the increase in linewidth in Fig. 3 (a) in the Main Text.

References

1. Rippard, W. H. et al. Spin-transfer dynamics in spin valves with out-of-plane magnetized CoNi free layers. *Phys. Rev. B* **81**, 014426 (2010).
2. Deac, A. M., et al. Bias-driven high-power microwave emission from MgO-based tunnel magnetoresistance devices. *Nat. Phys.* **4**, 308 (2008).

3. Maehara, H., et al. Large Emission Power over 2 μW with High Q Factor Obtained from Nanocontact Magnetic-Tunnel-Junction-Based Spin Torque Oscillator, *Appl. Phys. Express* **6**, 113005 (2013).

4. Fowley, C., et al. Zero-field spin-transfer oscillators combining in-plane and out-of-plane magnetized layers. *Appl. Phys. Express* **7**, 043001 (2014).

5. Lee, K. J., Deac, A. M., Redon, O., Nozieres, J. P., & Dieny, B. Excitations of incoherent spin-waves due to spin-transfer torque. *Nat. Mater.* **3**(12), 877 (2004).
